# Body composition in older acute stroke patients after treatment with individualized, nutritional supplementation while in hospital

**DOI:** 10.1186/1471-2318-10-75

**Published:** 2010-10-18

**Authors:** Lisa Ha, Truls Hauge, Per Ole Iversen

**Affiliations:** 1 Department of Internal Medicine, Østfold Hospital Trust Fredrikstad, 1603 Fredrikstad, Norway; 2 Department of Gastroenterology, Oslo University Hospital, Ullevål, 0407 Oslo, Norway; 3 Department of Nutrition, Institute of Basic Medical Sciences, PO Box 1046 Blindern, University of Oslo, 0316 Oslo, Norway; 4 Department of Hematology, Oslo University Hospital, Ullevål, 0407 Oslo, Norway

## Abstract

**Background:**

Individualized, nutritional support reduced undernutrition among older stroke patients and improved quality of life in our recent randomized, controlled trial. Weight control thus seems to be important after stroke, and methods for monitoring nutritional status need to be simple and non-invasive. Here we aimed to assess if the nutritional intervention altered body composition in men and women in this study cohort, and also to examine the correlation between the methods for assessing body-, fat- and fat-free mass.

**Methods:**

Acute stroke patients > 65 years at nutritional risk were randomized to either individualized, nutritional treatment with energy- and protein rich supplementation (intervention, n = 58) or routine, nutritional care (control, n = 66) while in hospital. Body composition was assessed with anthropometry and bioelectrical impedance. The follow-up period was three months.

**Results:**

During the first week while in hospital, weight loss was smaller in the intervention group compared with the controls (P = 0.013). After three months weight- and fat loss were significant in both men and women. Whereas no significant differences were found in changes in body composition between the male study groups, in the women both weight loss (P = 0.022) and fat loss (P = 0.005) was smaller in the intervention group compared with the controls. A high correlation (r = 0.87) between mid upper arm circumference (MUAC) and body mass index (BMI) was found.

**Conclusions:**

Individualized nutritional support to older stroke patients in hospital was beneficial for maintaining an adequate body mass and body composition the first week and seemed to have a preventive effect on fat loss among women, but not among men after three months. Measurement of MUAC may be used in the assessment of nutritional status when BMI cannot be obtained.

**Trial registration:**

This trial is registered with ClinicalTrials.gov, number NCT00163007.

## Background

In older persons, the presence of chronic diseases, polypharmacy, eating difficulties and various functional disabilities can result in inadequate dietary intake and malnutrition. Hence, protein- and energy undernutrition may be manifest or in progression when the older person is hospitalized for an acute stroke. Dysphagia or other feeding problems arising from neurological, cognitive or motoric impairments after stroke can give further nutritional deterioration [1-4]. Prospective cohort studies have shown a decrease in body weight after acute stroke and a loss of muscle- and fat mass [5,6]. When signs of protein- and energy deficits are present in acute stroke patients, there is an increased risk of poor functional outcome, pneumonia and other infections, gastrointestinal bleeding, bedsores and higher mortality [1,2,7-10]. This could in turn increase hospital stay, decrease quality of life and impair rehabilitation outcome. Hence, the main purpose of nutritional treatment after an acute stroke is to prevent or treat complications from energy- and protein undernutrition [11]. However, the impact of nutritional supplementation on body composition in elderly stroke patients is unclear. Randomized, controlled trials have not shown any differences in changes in body weight, triceps skinfold thickness or mid-upper arm circumference in stroke patients after supplementation with energy- and protein rich sip feedings compared to routine, nutritional care [12,13].

We have recently reported the results from a randomized, controlled trial in acute stroke patients more than 65 years at nutritional risk, and we observed that an individualized energy- and protein supplementation during hospital stay improved health-related quality of life and grip strength after three months [14]. Notably, this intervention also reduced clinically relevant weight loss (i.e. ≥ 5% in three months) in the intervention group compared with the controls although statistical significance was not reached (P = 0.055). Maintaining body weight is essential among acute stroke patients to prevent additional morbidity [11]. In order to achieve weight control and maintain energy- and protein status, it is important to understand how nutritional intervention affects body composition. Moreover, simple, quick and non-invasive methods for nutritional assessment are essential for monitoring nutritional status.

Hence, we examined the alterations in body composition using anthropometric measurements and bioelectrical impedance analysis in patients given individualized, nutritional support compared with those given routine care in our randomized trial. Gender-specific analyses were performed. We also examined the correlation between the different methods for assessing body-, fat- and fat-free mass.

## Methods

### Inclusion process

Between May 2005 and December 2007 stroke patients were consecutively enrolled from the medical acute care ward at Østfold Hospital Trust to which acute stroke patients more than 65 years in Østfold County, South-eastern Norway were referred. The initial choice of antithrombotic treatment in patients with ischemic stroke was acetylsalicylic acid. All patients underwent computer tomography scanning or magnetic resonance imaging. Those with ischemic stroke or cerebral haemorrhage were eligible for nutritional risk assessment. The patient was excluded from study entry either if the stroke diagnosis could not be confirmed or if the patient was critically ill, had severe dementia, could not be weighed or if there was planned discharge within 24 hours after the first visit by the trial assessor. Nutritional risk status was assessed within seven days after admission using the Malnutrition Universal Screening Tool (MUST) [15] with minor modifications adjusted for elderly patients, i.e. the cut-off value for body mass index (BMI) was set at ≤ 20 kg/m^2^. A marker of nutritional risk was either BMI ≤ 20 kg/m^2^, or unintentional weight loss of ≥ 5% the previous 3-6 months, or poor nutritional intake for at least five days, or the risk of inadequate nutritional intake for the next five days. Patients with at least one marker of nutritional risk present were included.

The inclusion process, sample size estimation and the randomization process is described in detail elsewhere [14]. In brief, we assessed 344 acute stroke patients with the MUST; 186 patients were at nutritional risk of which 16 refused to participate. We finally randomized 170 patients, and excluded three in the intervention group (not able to weigh (n = 1), non-stroke (n = 1) and withdrawn (n = 1)) and two in the control group (not able to weigh (n = 1) and withdrawn (n = 1)) after randomization. The remaining 165 patients were randomized median 3 (range 1-6) days after hospital admission (i.e. baseline) to either individual, nutritional treatment or routine care. After median 93 days (range 67-133 days) (i.e. three month follow-up) 124 patients were reassessed and completed the study. Twenty-two patients died and 19 patients refused to attend three month follow-up. Among those which completed the study, 91 had been reassessed median 8 days (range 3-16 days) (i.e. week 1) after study entry. Informed consent was obtained from all patients. If the patient was unable to give written consent due to impaired functional ability, a third person (from the family or nursing staff) was asked to confirm the patient's consent.

Sample power calculations, based on the intention to reduce the percentage of patients becoming undernourished (≥ 5% weight loss) from 30 to 10 [16], showed that 124 patients in total were required. The patients were randomized to individualized, nutritional treatment (intervention) or to routine care (control) using sequentially numbered envelopes containing the name of the allocated study group. The sequence of treatment allocation was prepared from a computer-generated randomisation list by a person not involved in patient assessments.

The study protocol was approved by The Regional Committee for Medical and Health Research Ethics, Health Region South of Norway. This study is registered with ClinicalTrials.gov, number NCT00163007.

### Intervention

The main treatment goal in the intervention group was to maintain or improve body weight during hospital stay. Nutritional treatment given was energy- and protein enriched meals, or established oral energy- and protein rich sip feedings (with 0.8-1.5 kcal and 0.04-0.1 g protein per mL), or enteral tube feeding (with 1.0 kcal and 4.0 kcal per mL) according to the estimated individual nutritional intake and nutritional needs calculated according to the Schofield equations [17]. Dietary recording and the intervention are described in detail elsewhere [14]. In brief, dietary intake was recorded by the nursing staff using standardized dietary registration forms. An individual, nutritional treatment plan was prepared for each patient describing type, amount and route of feeding. Energy- and protein enriched meals were given and oral sip feedings were prescribed on the medicine chart to 47 patients, and tube feeding was offered to a total of 11 patients with severe dysphagia. The patients in the intervention group were discharged with nutritional advice given by a dietitian to prevent undernutrition.

The nutritional management in the control group conformed to routine practice, i.e. with no further assessment of nutritional intake or needs and treated without an individualized nutritional plan. The patients were reviewed after one week if they still were in hospital and then three months after study entry. They were not contacted before the three month follow-up.

### Body composition analyses

Weight was measured on an electronic chair scale (SECA, Germany) to the nearest 0.1 kg. Height was estimated from the knee-heel length using the age and gender specific equations from Chumlea et al [18]. Multifrequency bioelectrical impedance spectroscopy (BIS) (Bodyscout, Fresenius, Germany) was used to determine extracellular water (ECW) and intracellular water (ICW) and to estimate fat mass, fat-free mass, lean tissue mass and body cell mass. The measurement was undertaken according to the manual for the BIS device. The patient had to remove all metal from the clothing and body, and be in a supine position for at least 10 minutes, and fasting for two hours, and not be engaged in heavy activity prior to the measurement. Patients with cardiac pacemakers or metallic stents were excluded from this measurement. Electrodes were attached to specified positions on the dorsal surface of the hand and the foot, either on the dominant or non-paretic side. During the measurement the patient was in a supine position with the arms and legs abducted from the body and instructed not to move or speak. Bioelectrical impedance analysis was not performed in 27% of the patients because of the exclusion criteria for the procedure, e.g. having a pacemaker. Mid upper arm circumference (MUAC) was measured with a tape measurer from the midpoint on the triceps on the dominant or non-paretic arm between the acromion and the olecranon processes. Triceps skinfold thickness (TSF) was measured at the midpoint with a skinfold caliper (Harpenden, Baty International, England) to the nearest 0.2 cm. We used the average of three measurements. Arm muscle circumference (AMC) was estimated from the formula: MUAC - (0.314 × TSF).

All clinical assessments were performed by one of two trial assessors. There were written procedures to standardise the measurements. The assessors were not blinded to which treatment the patient was assigned at study entry. To minimize the possible bias from not blinding at baseline, the information about the allocated treatment was made inaccessible to the assessor at three month follow-up.

### Statistical analyses

Between-group comparison for continuous variables was evaluated with Student's t test or Mann-Whitney *U *test. Normality was evaluated by the shape of the frequency histogram, normal Q-Q plot and Kalmogorov-Smirnov test. Comparison of weight change between the study groups was adjusted for baseline weight using a multiple regression analysis, and similar analysis were performed with BMI change. Within-group changes were compared with paired t-tests. The association between continuous variables is presented with Pearson's correlation coefficient r. Receiver operating characteristic (ROC) curve analysis was used to evaluate the accuracy of MUAC as a test to distinguish between BMI below or above a defined cut-off value. A P value < 0.05 was considered statistically significant. Statistical analyses was performed with the software package SPSS (version 16, SPSS Inc. Chicago, IL, USA).

## Results

The baseline characteristics in the study groups are shown in Table 1. The mean age was 79 years in those who completed the study, and there was no significant difference in age, percentage males and females, neurological status (Scandinavian Stroke Scale) or functional status (Barthel Index) between the two study groups. When we compared men and women, the baseline data did not significantly differ, except for age and prevalence of diabetes. The mean age in the men was significantly lower than in the women (77.3 vs 80.8 years), and there was significantly more diabetes in the men compared with the women (32% vs 19%). Both the energy (P = 0.005) and protein (P = 0.001) intake after one week's intervention was significantly higher in the female intervention group compared with the female controls (Table 2). In the males, energy and protein intake did not differ significantly between those in the intervention group and the controls.

**Table 1:** Baseline characteristics of the two study groups.

	Control (n = 66)	Intervention (n = 58)
Age, mean (SD) years	79.7 (6.8)	78.5 (7.4)
Males, number (%), females, number (%)	35 (53), 31 (47)	25 (43), 33 (57)
Cerebral haemorrhage, number (%)	8 (12.1)	4 (6.9)
Scandinavian Stroke Scale, median score (range)	42 (7-58)	41 (6-58)
Barthel index, median score (range)	11 (0-20)	11 (0-20)
Smoker,%	19	17.2
Diabetes,%	24.2	25.9
Albumin, mean (SD) g/L	39.1 (3.8)	39.5 (3.0)
Transferrin, mean (SD) g/L	2.1 (0.4)	2.2 (0.5)

There were no significant differences in baseline values between the study groups.

**Table 2:** Energy and protein intake during the first week after study entry in the male and the female study groups.

	Men			Women		
	Control	Intervention	P	Control	Intervention	P
Energy (kJ/kg)	69 (21)	76 (26)	0.34	59.7 (19.0)	83.2 (31.3)	0.005
Protein (g/kg)	0.79 (0.28)	0.78 (0.27)	0.87	0.65 (0.23)	0.88 (0.32)	0.001

Data are presented as mean (SD) intake per kg body weight.

### Changes in body composition assessed with bioelectrical impedance

The baseline values of body composition in both the male study groups and the female study groups were similar (Table 3). Both the female control group and the female intervention group had a mean fat loss of 3.6 kg (P < 0.001) and 1.4 kg (P = 0.005), respectively, which corresponded to 12.3% and 6.2% fat loss. The loss of fat mass was higher in the female control group compared with the female intervention group (P = 0.005). The mean fat loss was significant within both male study groups; 2.9 kg (P < 0.001) in the controls and 3.2 kg (P = 0.001) in the intervention group, but it did not differ between the study groups.

**Table 3:** Baseline values and changes after three months in bioelectrical impedance analysis of body composition in the male and the female study groups.

		Men			Women		
		Control (n = 26)	Intervention (n = 19)	P	Control (n = 23)	Intervention (n = 23)	P
Fat mass (kg)	Baseline	21.8 (8.0)	19.7 (8.4)	0.40	26.8 (11.0)	24.1 (7.0)	0.31
	%	28.1 (7.0)	27.0 (4.9)	0.64	38.3 (8.5)	37.2 (8.5)	0.57
	∆	-2.9 ± 0.6^a^	-3.2 ± 0.8^a^	0.69	-3.6 ± 0.6^a^	-1.4 ± 0.4^a^	0.005
	∆,%	-13.0 ± 2.7	-12.3 ± 5.7		-12.3 ± 2.3	-6.2 ± 1.7	
Fat-free mass (kg)	Baseline	53.9 (5.4)	51.2 (7.7)	0.17	40.6 (5.7)	39.5 (5.0)	0.47
	%	71.9 (7.0)	73.0 (8.3)	0.64	61.6 (8.5)	62.8 (5.6)	0.57
	∆	0.2 ± 0.6	0.8 ± 1.0	0.60	0.4 ± 0.6	0.03 ± 0.6	0.69
	∆,%	0.6 ± 1.0	2.1 ± 1.9		1.2 ± 1.5	0.1 ± 1.4	
Body cell mass (kg)	Baseline	28.1 (3.6)	27.0 (4.9)	0.38	19.6 (2.9)	19.1 (2.7)	0.52
	%	37.5 (4.7)	38.5 (5.9)	0.38	29.9 (4.8)	30.4 (3.1)	0.67
	∆	-0.1 ± 0.4	0.1 ± 0.7	0.77	0.3 ± 0.4	-0.03 ± 0.4	0.51
	∆,%	0.2 ± 1.4	1.5 ± 2.4		2.2 ± 2.0	0.1 ± 1.9	
Lean tissue mass (kg)	Baseline	48.9 (5.3)	46.8 (7.4)	0.28	34.9 (4.5)	34.4 (4.0)	0.65
	%	65.3 (7.6)	66.9 (9.7)	0.53	53.3 (8.6)	54.8 (5.6)	0.48
	∆	-0.1 ± 0.6	0.2 ± 0.9	0.76	0.4 ± 0.5	-0.1 ± 0.5	0.48
	∆,%	0.1 ± 1.2	1.1 ± 2.0		1.6 ± 1.5	-0.1 ± 1.5	
Total body water (L)	Baseline	38.7 (4.3)	36.7 (6.0)	0.21	29.7 (4.5)	28.7 (4.0)	0.40
	%	51.5 (4.9)	52.2 (5.1)	0.63	45.1 (5.8)	45.6 (4.0)	0.71
	∆	0.02 ± 2.4	0.5 ± 3.7	0.57	0.3 ± 2.3	0.01 ± 2.3	0.70
	∆,%	0.3 ± 1.2	2.2 ± 2.2		1.4 ± 1.7	0.1 ± 1.6	
Intracellular water (L)	Baseline	21.1 (2.9)	20.3 (3.7)	0.42	15.8 (2.4)	15.3 (2.2)	0.45
	%	28.1 (3.1)	28.9 (3.5)	0.45	24.0 (2.9)	24.4 (2.0)	0.64
	∆	-0.5 ± 1.9	-0.3 ± 2.1	0.86	0 ± 1.3	-0.2 ± 1.3	0.69
	∆,%	-1.5 ± 1.5	-0.9 ± 2.2		0.4 ± 1.8	-0.9 ± 1.7	
Extracellular water (L)	Baseline	17.5 (1.7)	16.4 (2.5)	0.084	13.8 (2.1)	13.3 (1.9)	0.37
	%	23.4 (2.3)	23.3 (2.2)	0.96	21.0 (2.9)	21.2 (2.1)	0.79
	∆	0.5 ± 0.2	0.9 ± 0.4	0.41	0.3 ± 0.2	0.2 ± 0.2	0.76
	∆,%	3.0 ± 1.3	6.1 ± 2.5		2.4 ± 1.8	1.3 ± 1.7	

Baseline values are presented as mean (SD). Change is presented as mean ± SEM. According to the manual of the BIS device "fat-free mass" comprises bone, skin, organs, blood, muscles, excess fluid and adipose water, "fat mass" comprises adipose lipids and essential lipids, "lean tissue mass" comprises bone, skin, organs, blood and muscles and "body cell mass" comprises skin, organs, blood and muscles. ∆: change from baseline to three months. ^a^Significant change within the group.

### Changes in anthropometry

**Figure 1:**
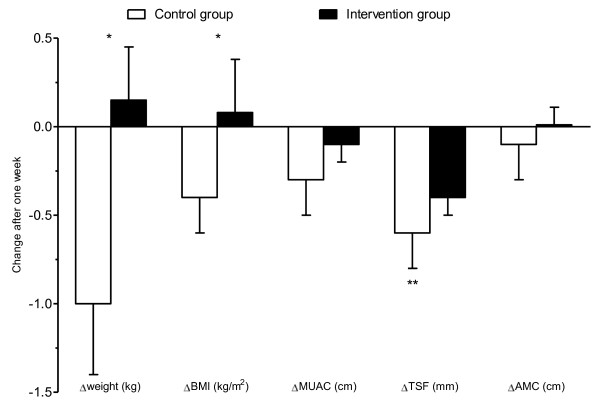
**Change (mean ± SEM) in anthropometry during the first week after study inclusion in the study groups**. ∆: Change. *Significantly different between the groups; **Significant change from baseline.

Figure 1 shows the changes in weight, BMI, MUAC, TSF and AMC after one week of intervention. One week after baseline the weight loss in the control group was significantly higher than in the intervention group (P = 0.013).

The baseline values and the changes after three months in anthropometry among men and women are presented in Table 4. Among the women the weight loss was significantly higher in the controls compared with the intervention group (P = 0.022). As for the men the mean weight loss was significant within both study groups, but was not different between the study groups. There was a significant decrease in TSF in both the male and female study groups, and this confirms that fat mass was decreased, but the change in TSF did not differ significantly between the study groups.

**Table 4:** Baseline values and changes in anthropometry after three months in the male and the female study groups.

		Men			Women		
		Control (n = 35)	Intervention (n = 25)	P	Control (n = 31)	Intervention (n = 33)	P
Weight (kg)	Baseline	76.4 (12.9)	72.4 (13.3)	0.25	68.3 (15.8)	61.7 (12.0)	0.061
	∆	-2.5 ± 0.7^a^	-2.0 ± 0.8^a^	0.66	-2.9 ± 0.7^a^	-0.8 ± 0.6	0.022
	∆,%	-2.9 ± 0.9	-2.6 ± 1.0		-4.2 ± 1.0	-1.1 ± 1.0	
BMI (kg/m^2^)	Baseline	25.3 (3.7)	24.5 (3.8)	0.42	27.2 (5.3)	24.5 (4.1)	0.026
	∆	-0.9 ± 0.2^a^	-0.8 ± 0.3^a^	0.84	-1.2 ± 0.3	-0.3 ± 0.2	0.052^b^
	∆,%	-3.0 ± 0.9	-2.9 ± 1.0		-4.2 ± 1.0	-1.1 ± 1.0	
MUAC (cm)	Baseline	29.8 (2.9)	30.1 (3.0)	0.71	29.6 (4.7)	27.7 (4.2)	0.094
	∆	-0.8 ± 0.2^a^	-1.0 ± 0.4^a^	0.56	-0.8 ± 0.3^a^	-0.3 ± 0.2	0.14
	∆,%	-2.5 ± 0.7	-3.2 ± 1.1		-2.5 ± 1.1	-0.8 ± 0.8	
TSF (mm)	Baseline	11.8 (3.5)	11.8 (3.4)	0.99	18.0 (7.2)	16.4 (5.4)	0.33
	∆	-0.9 ± 0.3^a^	-0.9 ± 0.4^a^	0.89	-1.6 ± 0.6^a^	-1.1 ± 0.5^a^	0.47
	∆,%	-6.0 ± 2.6	-6.0 ± 3.2		-5.8 ± 3.7	-4.8 ± 2.7	
AMC (cm)	Baseline	26.1 (2.3)	26.4 (2.5)	0.63	23.9 (3.1)	22.5 (3.0)	0.069
	∆	-0.5 ± 0.2^a^	-0.7 ± 0.3^a^	0.52	-0.3 ± 0.3	0.1 ± 0.2	0.23
	∆,%	-1.9 ± 0.7	-2.6 ± 1.0		-1.2 ± 1.1	0.4 ± 0.9	

Baseline values are presented as mean (SD). Change is presented as mean ± SEM. ∆: change from baseline to three months. ^a^Significant change within the group; ^b^Adjusted for baseline BMI.

### Correlation between baseline anthropometry and bioelectrical impedance

**Figure 2:**
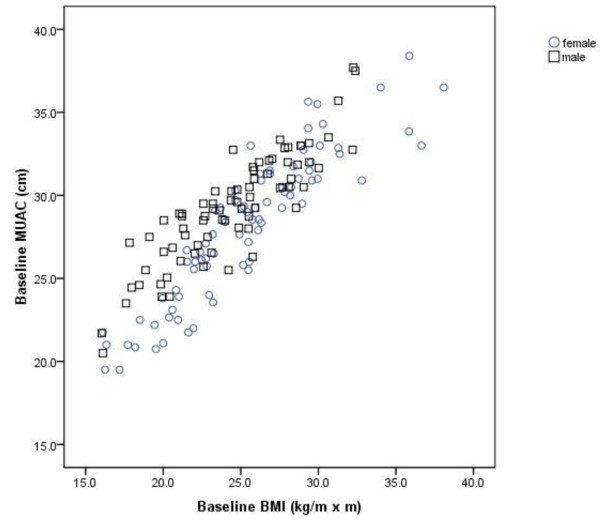
**Scatterplot of paired values for baseline BMI and baseline MUAC**. The corresponding Pearson correlation coefficient was r = 0.87 for all patients (n = 157), and r = 0.91 for women and r = 0.88 for men.

For both genders pooled TSF correlated well with fat mass (r = 0.68, P < 0.001), and AMC correlated well with fat-free mass (r = 0.68, P < 0.001), at baseline. Hence, there was a high correlation between body composition assessed with anthropometry and bioelectrical impedance. Moreover, there was a strong correlation between MUAC and BMI (r = 0.87, P < 0.001) for both genders pooled. The corresponding correlation for men and women analyzed separately, was r = 0.88 (P < 0.001) and r = 0.91 (P < 0.001), respectively (Figure 2).

### The association between MUAC and BMI

**Figure 3:**
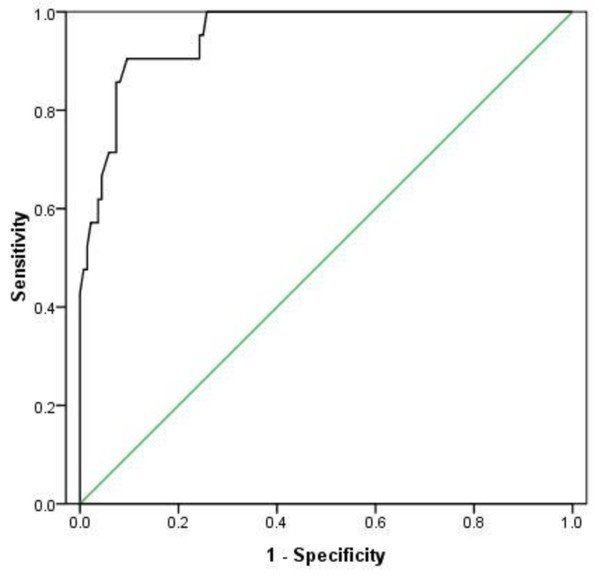
**Receiver operating characteristic (ROC) curve**. For each cut-off point for mid-upper arm circumference (MUAC) as a test to distinguish between BMI ≤ 20 kg/m^2 ^and BMI > 20 kg/m^2^, the values for the sensitivity and 1-specificity is plotted. Area under the curve (AUC) = 0.95 (P < 0.001).

We observed that MUAC was significantly associated with BMI independent of age and sex (P < 0.001). Given the high correlation (r = 0.87) between baseline MUAC and BMI, we wanted to explore if MUAC could be used as a test to distinguish between those with BMI ≤ 20 kg/m^2 ^(n = 21) and those with BMI > 20 kg/m^2 ^(n = 136). Sensitivity was plotted against 1-specificity for each measured value of MUAC generating a receiver operating characteristic (ROC) curve (Figure 3). The area under the ROC curve was 0.95 (95% CI 0.92 to 0.99) implying a high accuracy of the test. A cut-off value for MUAC was chosen to maximize the sensitivity and the specificity of the test. Therefore MUAC ≤ 25.5 cm was set to detect BMI ≤ 20 kg/m^2 ^(i.e. positive test) and MUAC > 25.5. cm was set to detect BMI > 20 kg/m^2 ^(i.e. negative test). Sensitivity, specificity, and positive predictive value (PPV) and negative predictive value (NPV) of the test were 0.91, 0.90, 0.59 and 0.98, respectively (Table 5). Consequently 91% of the patients with a BMI ≤ 20 kg/m^2 ^had a positive MUAC test and 90% of those with BMI > 20 kg/m^2 ^had a negative MUAC test. Among those with a positive MUAC test, 59% were placed in the correct BMI category, and the corresponding number in those with a negative MUAC test was 98%. Among the 13 patients with a false positive MUAC test, seven had a BMI ranging from 20.2 to 21.0 kg/m^2^.

**Table 5:** The accuracy of the "MUAC test" to distinguish between BMI ≤ 20 kg/m^2 ^and BMI > 20 kg/m^2^.

	MUAC cut-off	Sensitivity (95% CI)	Specificity (95% CI)	PPV (95% CI)	NPV (95% CI)
Both men and women	25.5 cm	0.91 (0.84-0.97)	0.90 (0.86-0.95)	0.59 (0.52-0.67)	0.98 (0.96-1.0)
Women	25.5 cm	1.0	0.86 (0.80-0.91)	0.52 (0.45-0.60)	1.0
Women	22.6 cm	0.91 (0.85-0.97)	0.96 (0.91-1.0)	0.77 (0.68-0.86)	0.99 (0.96-1.0)
Men	25.5 cm	0.80 (0.74-0.86)	0.96 (0.92-0.99)	0.73 (0.66-0.80)	0.97 (0.94-1.0)
Men	27.3 cm	0.90 (0.83-0.97)	0.83 (0.75-0.92)	0.45 (-0.07-0.56)	0.98 (0.94-1.0)

MUAC: mid-upper arm circumference; PPV: positive predictive value; NPV: negative predictive value.

A gender-specific analysis showed that a positive MUAC test set at ≤ 25.5 cm would give a low PPV of 0.52 in women and a high PPV of 0.73 in men (Table 5). If a positive MUAC test was set at ≤ 22.6 cm for women the PPV would increase to 0.77 and sensitivity, specificity and NPV would remain high.

## Discussion and Conclusions

This study has shown that in older acute stroke patients at nutritional risk which received individualized nutritional support during hospital stay, the loss of body weight was reduced after one week compared with control patients who received only routine, nutritional care. After three months the loss of body weight and fat mass was significant in both study groups, and in both men and women. However, the female intervention group had a significantly smaller loss of weight and fat mass compared with the female control group. This was not observed among the men. The estimation of fat and fat-free mass correlated well between bioelectrical impedance analysis and anthropometry.

We have previously shown that the intake of energy was 14% higher and significantly increased in the intervention group compared with the control group while in hospital [14], which could explain the better weight control after one week in the intervention group. Resting energy expenditure measured with indirect calorimetry after stroke was 88 kJ per kg body weight [19], and in immobile older patients, 84 kJ per kg body weight can be used to estimate the resting energy expenditure [20]. However, only the female intervention group in our study managed to reach a mean intake of 83 kJ and 0.9 g protein per kg body weight during hospital stay and was significantly higher than in the control group. Neither male study groups managed to achieve an energy intake above 80 kJ per kg body weight, and the intake did not differ between the groups. The lean tissue mass in men was 12 kg higher than in women, and it might have been more difficult for men to consume sufficient energy because the metabolic needs were higher. The higher prevalence of diabetes in the men compared with the women could possibly result in lower intake of food and drinks with added sugar, and hence, in the total intake of energy. Although we do not have data of the nutritional intake after hospital discharge, we observed that the women in the intervention group had a smaller loss of fat mass than the controls after three months indicating that the nutritional intake remained better in the female intervention group. This could have been attributed to the improved nutritional intake during hospital stay and the nutritional advice given before hospital discharge. In our study there were minor and non-significant increases in whole-body fat-free mass, body cell mass and lean tissue mass after three months in both genders and in both study groups. A loss of muscle mass has been shown with dual-energy X-ray absorptiometry (DXA) in both the paretic leg and the non-paretic in stroke patients who had not relearned to walk after two months, and after one year the lean mass in the non-paretic leg was regained [21]. In a study with 11 stroke patients there were no significant change over time in the muscle mass of the arms or legs [22].

In our study population mean baseline fat-free mass level was between the 25^th ^and 50^th ^percentile, and fat mass level between the 50^th ^and the 75^th ^percentile based on reference values adjusted for age and gender from a cohort of Swedish elderly [23]. Fat mass is lower and fat-free mass higher in men than in women, and fat mass naturally increases with age. Metabolic needs does not seem to be increased after an acute stroke [24]. However, feeding difficulties is not uncommon after an acute stroke [2] and may result in inadequate nutritional intake. Thus, the depletion of body fat as shown with both bioelectrical impedance analysis and with skinfold measurements in our study patients after three months, might have been related to partial starvation. During simple starvation glycogen reserves in the liver are quickly depleted during the first 24 hours of fasting. Muscle amino acids are mobilised and converted to glucose in the liver. However, muscle protein cannot continue to provide glucose substrates if fasting is prolonged for more than a few days, because the body would soon be depleted of essential proteins [25]. The mobilisation of free fatty acids (FFA) from adipose tissue is increased. When FFA are released from fat tissue to meet the energy needs of muscle tissue, this eventually leads to weight loss.

A low BMI is a marker of nutritional risk, and BMI is included in all nutritional screening tools recommended by the European Society for Parenteral and Enteral Nutrition (ESPEN) [26] to detect patients who could benefit from nutritional treatment. In clinical practice it requires more resources and time to weigh stroke patients who are bedridden compared with more able-bodied patients. Taking into account that is it more challenging to obtain weight and height in immobile patients, a measure of BMI may not be obtainable. We found a strong correlation (r = 0.87) between MUAC and BMI. With a cut-off point for MUAC at 25.5 cm, 80% and 96% of the men would be correctly categorized as underweight or not underweight, respectively. Among men with a positive MUAC test 73% would be underweight and 97% of those with a negative MUAC test would be not underweight. For women a positive test set at MUAC ≤ 22.6 cm would give the corresponding numbers of 91%, 96%, 77% and 99%. By using such a "MUAC for BMI test", more stroke patients could be assessed for nutritional risk in clinical practice. However, the validity of using MUAC to identify those patients which would benefit from nutritional treatment, needs to be confirmed in larger studies.

The study groups were well-balanced for most variables at baseline except for weight and BMI. Thus, when we compared changes in weight and BMI in the groups, the data analyses were adjusted for the respective baseline values. The patients in the intervention group with missing bioelectrical impedance data were relatively younger and weighed less. The mortality rate was 13%, and 12% of the patients did not participate in follow-up after three months, which is considerably lower than the reported loss to follow-up of 21-54% of elderly subjects participating in non-pharmacological trials [27-30].

Bioelectrical impedance analysis provides a validated and relatively easy and non-invasive approach to assess body composition among elderly patients and with minimal discomfort [23,31-33]. Whole body BIA allows the determination of the fat-free mass and TBW in subjects without significant fluid or electrolyte abnormalities and when using established procedures [34]. The determination of body fluid volumes via bioelectrical impedance methods is based on the assumption that electrical current at low frequencies cannot penetrate cell membranes and thus flows through the ECW space only, while high frequency current flows through both the ECW and ICW spaces [35]. Disturbances in fluid distribution can give errors in body fluid analyses and therefore in the estimation of fat and fat-free mass [32]. The TBW and ECW measured by BIS have been validated against reference methods (D_2_O dilution and NaBr dilution method, respectively) with good accuracy, in both healthy subjects and in patients with imbalanced fluid status [35]. Fat-free mass and fat mass estimates measured by a BIS device similar to the device used in our study have been validated against DXA estimates in a cohort of older subjects (average age 75 years). Average FFM measured by BIS was in agreement with DXA. However, there was a small systematic positive bias although a large individual variation was observed. Average fat mass measured by BIS was also in agreement with DXA, but with a small systematic negative bias [36]. However, there is a lack of validation studies in stroke patients specifically. To minimize potential errors associated with the bioelectrical impedance measurements, we provided standardised conditions (supine position, resting, fasting) for the measurements. Moreover, since we were mainly interested in the relative change in body composition among the individual patients during the three months observation period, any measurement error is less likely to alter our findings.

In summary, this study suggests that body composition is less negatively affected in older stroke patients receiving individualized nutritional support during hospital stay compared with routine, nutritional care. There is a deterioration in nutritional status after hospital discharge. Among women this nutritional intervention strategy reduced the catabolic process after three months. Measurement of MUAC may be used in assessment of nutritional status when BMI cannot be obtained. In older stroke patients at nutritional risk, we recommend regular assessment of dietary intake and nutritional status after hospital discharge as a part of follow-up in local health care.

## Competing interests

The authors declare that they have no competing interests.

## Authors' contributions

LH participated in the development and design of the study, conducted and supervised assessments and procedures within the study, had full access to the complete set of data and drafted the manuscript. TH and POI contributed to the study design, reviewed and interpreted data and participated in writing of the manuscript. All authors have seen and approved the final version of the manuscript.

## Pre-publication history

The pre-publication history for this paper can be accessed here:

http://www.biomedcentral.com/1471-2318/10/75/prepub
